# Factors associated with early receipt of COVID-19 vaccination and adherence to second dose in the Veterans Affairs healthcare system

**DOI:** 10.1371/journal.pone.0259696

**Published:** 2021-12-01

**Authors:** George N. Ioannou, Pamela Green, Emily R. Locke, Kristin Berry

**Affiliations:** 1 Division of Gastroenterology, Veterans Affairs Puget Sound Healthcare System and University of Washington, Seattle, WA, United States of America; 2 Research and Development, Veterans Affairs Puget Sound Health Care System, Seattle, WA, United States of America; Hualien Tzu Chi Hospital Buddhist Tzu Chi Medical Foundation, TAIWAN

## Abstract

**Background:**

We aimed to determine factors independently associated with early COVID-19 vaccination and adherence to two-dose regimens.

**Methods:**

Among persons receiving care in the Veterans Affairs (VA) healthcare system (n = 5,766,638), we identified those who received at least one dose of COVID-19 vaccination through the VA, during the first ~3months following emergency use authorization, from December 11, 2020 to March 9, 2021 (n = 1,569,099, or 27.2%, including 880,200 (56.1%) Moderna, 676,279 (43.1%) Pfizer-BioNTech and 12,620 (0.8%) Janssen vaccines).

**Results:**

Follow-up for receipt of vaccination began on December 11, 2020. After adjustment for baseline characteristics ascertained as of December 11, 2020, factors significantly associated with vaccination included older age, higher comorbidity burden, higher body mass index category, Black (vs. White) race (adjusted hazard ratio [AHR] 1.19, 95% CI 1.19–1.20), Hispanic (vs. non-Hispanic) ethnicity (AHR 1.12, 95% CI 1.11–1.13), urban (vs. rural) residence (AHR 1.31, 95% CI 1.31–1.31), and geographical region, while AI/AN race (vs. White), was associated with lower vaccination rate (AHR 0.85, 95% CI 0.84–0.87). Among persons who received both doses of Moderna or Pfizer-BioNTech vaccines, 95.3% received the second dose within ±4 days of the recommended date. Among persons who received the first vaccine dose, only 3.2% did not receive the second dose within 42 days for Pfizer versus 4.0% for Moderna (p<0.001). Factors independently associated with higher likelihood of missing the second dose included younger age (10.83% in 18–50 yo vs. 2.72% in 70–75 yo), AI/AN race, female sex, rural location, geographical region and prior positive test for SARS-CoV-2.

**Conclusions:**

We identified sociodemographic and clinical factors that may be used to target vaccination efforts and to further improve adherence to second vaccine dosing.

## Introduction

The Food and Drug Administration (FDA) issued Emergency Use Authorization (EUA) for COVID-19 vaccines by Pfizer-BioNTech on December 11, 2020, by Moderna on December 18, 2020, and by Janssen on February 27, 2021. The first two are administered as a 2-dose series, 3 weeks (Pfizer-BioNTech) or 4 weeks (Moderna) apart, while Janssen is a single-dose vaccine.

The Centers for Disease Control and Prevention (CDC) published recommendations on December 21, 2020 supporting a phased approach to vaccine allocation during the first few months after EUA: phase 1a (healthcare personnel and long-term care facility (LTCF) residents), phase 1b (frontline essential workers and persons aged ≥75 years), phase 1c (essential workers, persons aged 65–74 year and persons aged 16–64 years with high-risk medical conditions) and finally phase 2, which includes the remaining population [1]. These recommendations were adopted by the Veterans Affairs (VA) healthcare system, the largest comprehensive healthcare system in the United States, with some minor modifications to reflect the COVID-19 epidemiology of its population [2].

The extent to which older age-groups and persons with high-risk comorbid conditions were prioritized for vaccination in the first 3 months after EUA in real-world practice is unclear. Geographic variations in access to vaccination have been reported. Also, early reports suggested that racial/ethnic minority groups were under-represented among vaccinees in the United States, especially Hispanic, Black and American Indian/Alaska Native (AI/AN) groups [3], despite being disproportionately affected by COVID-19. Factors associated with receipt of different COVID-19 vaccines and adherence to the receipt and timing of the required second dose of Moderna and Pfizer-BioNTech vaccines are unclear. Differences in cold storage requirements of Pfizer-BioNTech (-70C) versus Moderna (-20C) or differences in perceived effectiveness or side effects of different vaccines may have resulted in associations of sociodemographic or clinical characteristics with use of a specific COVID-19 vaccine.

We aimed to describe factors associated with time-to-receipt of COVID-19 vaccination, type of vaccine used, and adherence to recommended two-dose regimens in the Veterans Affairs (VA) healthcare system, during the first 3 months of vaccine availability after EUA from December 11 to March 9, 2021.

## Methods

### Study setting and data source

The VA provides care at 168 medical centers and 1,053 outpatient clinics throughout the country and employs a comprehensive, nationwide electronic health records system (EHR). We used data from the VA’s Corporate Data Warehouse (CDW), a relational database of VA enrollees’ comprehensive EHR [4]. The CDW includes the “COVID-19 Shared Data Resource [CSDR]”, a set of analytic variables and datasets on all VA enrollees tested for or vaccinated against COVID-19 developed and maintained by the VA Informatics and Computing Infrastructure (VINCI) specifically to facilitate COVID-19 research and operations.

The study was approved by the VA Puget Sound Institutional Review Board. This study followed the Strengthening the Reporting of Observational Studies in Epidemiology (STROBE) reporting guideline.

### Study population and ascertainment of COVID-19 vaccination

We identified all VA enrollees who were alive as of December 11, 2020 and who had an inpatient or outpatient encounter in the VA healthcare system in the preceding 12 months or until March 9, 2021 (n = 5,766,638). We identified the subset of these VA enrollees who received at least one dose of an approved COVID-19 vaccine by the VA, on or after the EUA date of each vaccine, between December 11, 2020 (the date of EUA authorization for Pfizer-BioNTech) and March 9, 2021 (n = 1,569,101). Type and date of each vaccine dose were ascertained from VA pharmacy records. VA *employees*, including healthcare providers eligible for vaccination at the earliest phase 1a, were not included in the study.

### Baseline characteristics

We ascertained sociodemographic characteristics and comorbid conditions included in the CDC prioritization criteria or known to be associated with SARS-CoV-2 infection or adverse outcomes [5–17] in order to determine whether they were associated with receipt of vaccination. Baseline characteristics were ascertained as of December 11, 2021, the beginning of the follow-up period. These included age, sex, self-reported race (White, Black, Asian, American Indian/Alaska Native [AI/AN], Pacific Islander/Native Hawaiian [PI/NH], Other) and ethnicity (Hispanic, non-Hispanic), urban vs rural residence (based on zip codes, using data from the VA Office of Rural Health [18], which uses the Secondary Rural-Urban Commuting Area [RUCA] for defining rurality), geographic location (divided into 10 standard U.S. Federal Regions [19]), body mass index (BMI, using measured weight and height recorded prior to December 11, 2021), and comorbid conditions including diabetes, congestive heart failure (CHF), chronic obstructive pulmonary disease (COPD) and chronic kidney disease (CKD) defined by appropriate international classification of disease (ICD10) codes. These comorbid conditions were defined if the ICD10 codes were recorded in the 2-year period before December 11, 2020. We calculated the Charlson Comorbidity Index (CCI), using the Deyo modification [20–22], as a measure of comorbidity burden, using a 2-year window prior to December 11,2020. We identified persons who had a documented positive SARS-CoV-2 polymerase chain reaction (PCR) test in the VA healthcare system before December 11, 2020.

In order to better capture relevant geographic variability related to institutional factors, we additionally categorized persons according to the 19 VA administrative regions, known as Veterans Integrated Service Networks or VISNs [23]. Also, we categorized VA medical centers according to the proportion of vaccines administered that were manufactured by Moderna vs. Pfizer-BioNTech. Data on VA enrollees’ employment status (e.g. whether a person was a “frontline essential worker” or an “essential worker”) were not available.

Missing values for BMI (11.6%) were multiply imputed using values of the other characteristics listed above; all missing values were successfully imputed. “Missing” values for race or ethnicity included persons who refused to declare their race/ethnicity or reported unknown or mixed race/ethnicity and did not self-identify as belonging to one of the pre-specified racial or ethnic group. For these persons we did not perform imputation of race/ethnicity, but rather included them in a “missing/unknown/refused” category.

### Statistical analysis

Kaplan Meier curves were used to compare cumulative incidence of vaccination by selected characteristics. Cox proportional hazards regression stratified by VA medical center was used to identify factors independently associated with time-to-vaccination (receipt of first vaccine dose) after adjusting for age, sex, race, ethnicity, VISN, urban/rural residence, BMI and CCI. Time-to-vaccination analyses began on December 11, 2020 and were censored on March 9, or at the time of death if earlier. The proportional hazards assumption was assessed graphically using log(-log) plots. We determined adherence to the timing of the recommended second dose of Pfizer-BioNTech (21 ± 4 days) or Moderna (28 ± 4 days) and the proportion who failed to receive the second dose within the maximum allowed time (42 days). Logistic regression was used to identify factors associated with a missed second dose (defined as no second dose within 42 days of the first dose among persons who had at least 42 days of follow-up time) using logistic regression adjusting for the aforementioned characteristics plus type of vaccine (Moderna vs. Pfizer-BioNTech) and the percentage of all vaccinees vaccinated with Moderna at each medical center. Logistic regression was also used to identify factors independently associated with receipt of Moderna (vs. Pfizer-BioNTech) vaccine after adjusting for these characteristics.

VISN was used rather than federal region in the multivariable models because VISN captures VA-specific variability in distribution of vaccination more accurately.

## Results

### Baseline characteristics of VA enrollees

Mean age was 62.4 years, with a substantial proportion ≥65 (52.8%), ≥75 (23.0%) or ≥85 (7.21%) years of age (Table 1). The majority of VA enrollees were male (90.5%) and white (71.1%), with 17.74% of Black race and 6.92% of Hispanic ethnicity. Most cohort members (64.24%) had a CCI score of one or higher.

**Table 1:** Association of selected factors with receipt of at least one dose of COVID-19 vaccination among VA enrollees between December 11, 2020 and March 9, 2021.

	Number of VA enrollees (%) N = 5,766,638	Proportion Vaccinated 12/11/20 to 3/9/21	Vaccination rate (Per 100 person-months)	Unadjusted hazard ratio	Adjusted** hazard ratio
**All persons**	5,766,638 (100)	27.21	10.15	**N/A**	**N/A**
**Sex**					
Female	548,035(9.50)	16.64	5.88	1	1
Male	5,218,603(90.50)	28.32	10.63	1.79(1.78–1.80)	0.96 (0.95–0.96)
**Age (years)**					
18 to <50	1,342,095(23.27)	5.97	2.04	1	1
50 to <60	833,895(14.46)	16.46	5.77	2.90(2.88–2.93)	2.70 (2.68–2.72)
60 to <65	544,713(9.45)	24.78	8.87	4.49(4.45–4.53)	4.00 (3.96–4.03)
65 to <70	604,903(10.49)	35.97	13.62	7.25(7.19–7.31)	6.38 (6.33–6.44)
70 to <75	1,113,376(19.31)	38.33	14.84	7.96(7.90–8.02)	7.08 (7.02–7.13)
75 to <80	596,150(10.34)	45.74	19.24	10.96(10.87–11.05)	9.66 (9.58–9.74)
80 to <85	315,975(5.48)	41.98	17.46	9.73(9.64–9.81)	8.63 (8.55–8.71)
85 to <90	255,799(4.44)	41.59	17.68	9.79(9.70–9.88)	8.71 (8.62–8.79)
≥90	159,732(2.77)	37.96	16.12	8.63(8.54–8.73)	7.82 (7.74–7.91)
**Race**					
White	4,100,331(71.10)	27.77	10.42	1	1
Black	1,023,211(17.74)	26.52	9.77	1.00(1.00–1.01)	1.19 (1.19–1.20)
Asian	72,864(1.26)	22.03	7.96	0.79(0.78–0.81)	1.20 (1.18–1.22)
American Indian/Alaska Native	52,004(0.90)	19.69	7.13	0.70(0.69–0.71)	0.85 (0.84–0.87)
Pacific Islander/ Native Hawaiian	55,915(0.97)	24.09	8.84	0.85(0.84–0.87)	1.02 (1.00–1.03)
Declined/Unknown/Missing	462,313(8.02)	25.78	9.55	0.92(0.91–0.93)	0.96 (0.96–0.97)
**Ethnicity**					
Non-Hispanic	5,073,564(87.98)	27.44	10.25	1	1
Hispanic	399,202(6.92)	23.31	8.5	0.75(0.74–0.75)	1.12 (1.11–1.13)
Declined/Unknown/Missing	293,872(5.10)	28.57	10.66	1.03(1.02–1.04)	1.18 (1.17–1.19)
**Urban/Rural**					
Rural/Highly rural	2,940,158(50.99)	24.55	9.07	1	
Urban	2,786,387(48.32)	30.06	11.33	1.26(1.26–1.27)	1.31 (1.31–1.31)
Missing	40,093(0.70)	24.16	8.92	1.07(1.05–1.09)	0.98 (0.96–1.00)
**Geographical Federal Region** *					
1	229,522(3.98)	34.7	13.26	1.42(1.39–1.45)	1.30 (1.29–1.31)
2	334,925(5.81)	34.93	13.37	1.28(1.27–1.30)	1.25 (1.24–1.26)
3	537,931(9.33)	28.98	10.88	1.28(1.26–1.29)	1.14 (1.13–1.15)
4	1,489,186(25.82)	25.04	9.29	1	1
5	877,104(15.21)	29.65	11.15	1.10(1.09–1.11)	1.10 (1.10–1.11)
6	792,124(13.74)	22.58	8.3	1.11(1.10–1.13)	0.94 (0.94–0.95)
7	310,914(5.39)	31.34	11.98	1.36(1.34–1.38)	1.30 (1.29–1.31)
8	234,853(4.07)	24.89	9.22	1.24(1.22–1.27)	1.07 (1.06–1.08)
9	687,473(11.92)	26.86	9.94	1.18(1.16–1.19)	1.04 (1.03–1.04)
10	271,993(4.72)	23.62	8.66	1.17(1.15–1.19)	0.96 (0.95–0.96)
**VA Integrated Service Network (VISN)** [23]					
1	220,377(3.82)	34.58	13.2	1.02(1.02–1.03)	1.03 (1.02–1.04)
2	242,668(4.21)	34.54	13.18	1.02(1.02–1.03)	1.00 (0.99–1.01)
4	254,925(4.42)	34.36	13.23	1.03(1.02–1.04)	1.02 (1.01–1.02)
5	179,188(3.11)	27.04	10.1	0.77(0.77–0.78)	0.87 (0.86–0.87)
6	364,510(6.32)	23.4	8.56	0.65(0.64–0.65)	0.76 (0.75–0.76)
7	424,118(7.35)	20.56	7.46	0.56(0.56–0.57)	0.64 (0.64–0.65)
8	555,818(9.64)	33.33	12.84	1	1
9	251,631(4.36)	22.08	8.16	0.62(0.61–0.63)	0.69 (0.68–0.69)
10	442,131(7.67)	27.25	10.16	0.78(0.77–0.78)	0.78 (0.78–0.79)
12	238,310(4.13)	33.97	12.97	1.01(1.00–1.01)	1.01 (1.00–1.01)
15	221,137(3.83)	28.15	10.63	0.82(0.81–0.83)	0.90 (0.89–0.91)
16	388,049(6.73)	25.57	9.5	0.72(0.72–0.73)	0.81 (0.80–0.81)
17	392,083(6.80)	18.02	6.51	0.49(0.48–0.49)	0.60 (0.60–0.61)
19	284,050(4.93)	22.19	8.1	0.61(0.61–0.62)	0.73 (0.73–0.74)
20	271,729(4.71)	23.36	8.56	0.65(0.64–0.65)	0.75 (0.75–0.76)
21	293,539(5.09)	29.06	10.84	0.83(0.82–0.84)	0.87 (0.86–0.87)
22	452,543(7.85)	25.28	9.31	0.71(0.70–0.71)	0.79 (0.78–0.79)
23	289,826(5.03)	34.49	13.3	1.04(1.03–1.05)	1.12 (1.11–1.13)
**BMI (kg/m** ^**2**^ **)** †					
<18.5	43,926(0.76)	26.47	10.1	0.97(0.95–0.99)	0.80 (0.79–0.82)
18.5 to <25	1,068,389(18.53)	27.83	10.51	1	1
25 to <30 (Overweight)	2,045,494(35.47)	28.17	10.58	1.01(1.00–1.01)	1.09 (1.08–1.09)
30 to <35 (Obese I)	1,567,982(27.19)	26.87	9.97	0.95(0.95–0.96)	1.13 (1.12–1.13)
35 to <40 (Obese II)	702,482(12.18)	25.59	9.41	0.90(0.89–0.90)	1.14 (1.13–1.14)
≥40 (Obese III)	338,365(5.87)	24.49	8.94	0.85(0.85–0.86)	1.11 (1.10–1.12)
**Charlson Comorbidity Index (CCI)**					
0	2,062,422(35.76)	17.28	6.15	1	1
1	1,059,983(18.38)	23.49	8.57	1.42(1.41–1.43)	1.03 (1.03–1.04)
2	689,303(11.95)	30.1	11.34	1.91(1.90–1.92)	1.12 (1.12–1.13)
3	568,779(9.86)	33.63	12.9	2.20(2.19–2.22)	1.19 (1.18–1.20)
4	386,317(6.70)	36.66	14.31	2.47(2.46–2.49)	1.27 (1.26–1.28)
5–6	510,129(8.85)	39.63	15.77	2.77(2.76–2.79)	1.35 (1.35–1.36)
7–8	264,028(4.58)	43.05	17.59	3.15(3.13–3.17)	1.49 (1.48–1.50)
≥9	225,677(3.91)	47.64	20.38	3.72(3.69–3.75)	1.74 (1.72–1.75)
**Diabetes**					
No	4,243,267(73.58)	24.06	8.85	1	1
Yes	1,523,371(26.42)	35.98	13.99	1.66(1.65–1.66)	1.15 (1.14–1.15)
**Chronic Kidney Disease**					
No	5,237,522(90.82)	25.86	9.57	1	1
Yes	529,116(9.18)	40.56	16.47	1.83(1.82–1.84)	1.20 (1.19–1.20)
**Congestive heart failure**					
No	5,543,788(96.14)	26.56	9.87	1	1
Yes	222,850(3.86)	43.43	18	1.95(1.93–1.96)	1.32 (1.31–1.33)
**COPD**					
No	5,088,348(88.24)	25.78	9.55	1	1
Yes	678,290(11.76)	37.94	15	1.62(1.62–1.63)	1.17 (1.17–1.18)
**Tested Positive Before December 11, 2020**					
No	5,655,501(98.07)	27.21	10.15	1	1
Yes	111,137(1.93)	27	10.15	0.97(0.96–0.98)	1.05 (1.04–1.06)

*Categorized according to the 10 “Standard Federal Regions” drawn up by the Office of Management and Budget: 1 (CT, MA, ME, NH, RI, VT), 2 (NJ, NY, PR), 3 (DC, DE, MD, PA, VA, WV), 4 (AL, FL, GA, KY, MS, NC, SC, TN), 5 (IL, IN, MI, MN, OH, WI), 6 (AR, LA, NM, OK, TX), 7 (IA, KS, MO, NE), 8 (CO, MT, ND, SD, UT, WY), 9 (AZ, CA, GU, HI, NV), 10 (AK, ID, OR, WA).

** Simultaneously adjusted for age, sex, race, ethnicity, urban/rural location, BMI, CCI and VISN, and stratified by VA medical center. When looking at diabetes, COPD, CHF and CKD, we used a different model that did not simultaneously adjust for CCI since CCI includes these comorbidities.

† BMI was categorized using the World Health Organization groups [28].

### Factors associated with vaccination

**Fig 1:**
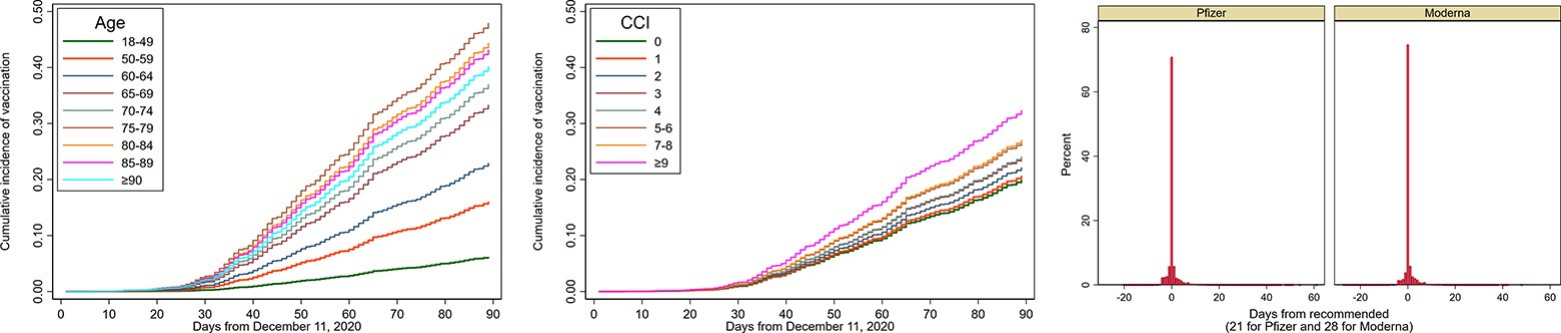
Cumulative incidence of receiving vaccination by age category (a) or CCI category (b) and timing of second vaccination dose (c). a. Kaplan-Meier cumulative incidence of vaccination from December 11, 2020 to March 9, 2021 by age group. b. Kaplan-Meier cumulative incidence of vaccination from December 11, 2020 to March 9, 2021 by Charlson Comorbidity Index (CCI) group. c. Distribution of the timing of the second doses of Pfizer-BioNTech and Moderna vaccines, shown as days from the recommended date (which is 21 days from the first dose for Pfizer-BioNTech and 28 days for Moderna).

As of March 9, 2021, 1,569,101 out of 5,766,638 (27.2%) VA enrollees received at least one vaccination dose through the VA, including 880,200 (56.1%) with Moderna, 676,279 (43.1%) with Pfizer-BioNTech and 12,620 (0.8%) with Janssen vaccines. Vaccination rates were very low for the first ~30 days after December 11 (Fig 1A and 1B), corresponding to CDC Phase 1a, when vaccination was restricted almost exclusively to healthcare workers not captured in our data and long-term care facility residents, but increased rapidly thereafter.

Graphical assessment of our Cox multivariable model using log(-log) plots suggested good adherence to the proportional hazards assumption as evidenced by near parallel plots (representative plots for CCI categories and CKD are shown in S1A and S1B Fig).

Vaccination rate was higher in men versus women (10.63 vs. 5.88 per 100 person-months); however, after adjustment for baseline characteristics, men were slightly less likely to undergo vaccination than women (adjusted hazard ratio [AHR] 0.96, 95% CI 0.95–0.96) (Table 1).

Vaccination rate increased dramatically with increasing age from 2.04 per 100 person-months in 18–50 year-olds to 19.24 per 100 person-months in 75–80 year-olds, and then declined slightly for older age groups. These associations persisted after adjustment.

Compared to White persons, Black and Asian persons had slightly lower vaccination rates, but were more likely to be vaccinated after adjustment for baseline characteristics (AHR 1.19, 95% CI 1.17–1.19 for Black and AHR 1.20, 95% CI 1.18–1.22 for Asian persons). Conversely AI/AN persons had lower vaccination rates than White persons (7.13 vs. 10.42 per 100 person-months), which persisted after adjustment (AHR 0.85, 95% CI 0.84–0.87). Compared to non-Hispanic persons, Hispanic persons had lower vaccination rates (8.5 vs. 10.25 per 100 person-months) but were more likely to receive vaccination after adjustment for baseline characteristics (AHR 1.12, 95% CI 1.11–1.13).

Vaccination rates differed by geographical region ranging from 8.3 per 100 person-months in region 6 (AR, LA, NM, OK, TX) to 13.37 per 100 person-months in region 2 (NY, NJ, PR); substantial associations between geographical region and vaccination persisted after adjustment for baseline characteristics. Also, vaccination rates varied significantly by VISN from 6.61 (VISN 17 in Texas) to more than 13 per 100 person-months in VISNs 1,2,4 and 23. Compared to persons residing in rural areas, those living in urban areas were more likely to undergo vaccination (AHR 1.31, 95% CI 1.31–1.31).

Compared to the “normal” BMI category (18.5–25 Kg/m^2^), persons in overweight and obese categories were slightly more likely to receive vaccination after adjustment for baseline characteristics. Higher CCI category was associated with significantly higher likelihood of vaccination, which was attenuated somewhat but persisted after adjustment for baseline characteristics. Diabetes, CKD, CHF and COPD were all significantly associated with vaccination. Persons who previously tested positive for SARS-CoV-2 had similar vaccination rates to those without a prior positive test and were slightly more likely to be vaccinated after adjustment for baseline characteristics (AOR 1.05, 95% CI 1.04–1.06).

### Timing and adherence to second vaccination dose

Among persons who received both vaccine doses as of March 9, 2021 (n = 851,144), 434,463 (51%) received the Pfizer and 416,681 (49.0%) the Moderna vaccine. Fig 1C shows the distribution of the second dose of vaccine relative to the recommended time of 21 days from the first dose for Pfizer-BioNTech and 28 days for Moderna. The second vaccine dose was administered within 21 ± 4 days in 95.3% of cases for Pfizer-BioNTech and in 28 ± 4 days in 95.4% for Moderna (Table 2). An additional, 4.1% received the Pfizer-BioNTech second dose from 26–42 days and 3.6% received the Moderna second dose from 33–42 days. Only a very small proportion (0.1% for Pfizer-BioNTech and 0.3% for Moderna) received the second dose “late”, i.e. beyond the allowable interval of 42 days. Also, a very small proportion (0.1%) received a second dose that was a different vaccine type from the first.

**Table 2:** Distribution of different COVID-19 vaccines administered by the VA healthcare system to VA enrollees from December 11, 2020 to March 9, 2021 and adherence to, and timing of the second dose of Pfizer and Moderna vaccinations among persons who received the first dose.

	Pfizer-BioNTech or Moderna	Pfizer-BioNTech	Moderna
**A. Timing of second vaccine dose among persons who received two doses**			
**Received ≥1 dose**	1,556,479	676,279(43.4)	880,200(56.6)
**Received 2 doses**	851,144	434,463(51.0)	416,681(49.0)
**Dosing interval**^**1**^ **among persons who received 2 doses**			
Early^2^	4,062(0.5)	1,787(0.4)	2,275(0.5)
During recommended interval^3^	811,443(95.3)	413,860(95.3)	397,583(95.4)
After recommended interval but within allowable interval^4^	33,056(3.9)	17,921(4.1)	15,135(3.6)
Late^5^	1,632(0.2)	437(0.1)	1,195(0.3)
Received second dose but with a different vaccine type (any time)	951(0.1)	458(0.1)	493(0.1)
**B. Completion status among persons with sufficient follow-up time to receive second dose** ^**6**^			
Number with sufficient time (≥42 days) to receive second dose	467,450	211,294	256,156
Missed second dose (i.e. no second dose up to 42 days)	16,973(3.6)	6,813(3.2)	10,160(4.0)
Received second dose up to 42 days	450,477(96.4)	204,481(96.8)	245,996(96.0)

^1^ The recommended dosing interval between first and second dose is 21 days for Pfizer-BioNTech and 28 days for Moderna.

^2^ Received second dose <17 days (Pfizer-BioNTech) or <24 days (Moderna) after first dose.

^3^ Received second dose 17–25 days (Pfizer-BioNTech) or 24–32 (Moderna) days after first dose.

^4^ Received second dose 26–42 days (Pfizer-BioNTech) or 33–42 (Moderna) days after first dose.

^5^Received second dose >42 days after first dose.

^6^Had at least 42 days of follow-up, ie received first dose on or before January 26, 2021.

Among persons who had at least 42 days of follow-up time, i.e. sufficient to receive the second dose within the maximum allowable interval, only 3.2% missed the second dose in that interval for Pfizer-BioNTech versus 4.0% for Moderna (p-value <0.001) (Table 2). Factors independently associated with lower likelihood of missing the second dose included older age (e.g. missed rate 2.72% in 70 to <75 year-old vs. 10.83% in 18 to <50 yo, adjusted odds ratio (AOR) 0.24, 95% CI 0.23–0.26) male sex, Black race, and urban location (Table 3). Factors independently associated with higher likelihood of missing the second dose included receiving Moderna (vs. Pfizer-BioNTech) (AOR 1.19, 95% CI 1.15–1.23), AI/AN race (AOR 1.34, 95% CI 1.14–1.57), having a prior positive SARS-CoV-2 test (AOR 1.39, 95% CI 1.27–1.53) and being at a medical center with high percentage Moderna use. There were also significant differences in second dose adherence by federal region or VISN (Table 3).

**Table 3:** Association of selected factors with missing the second vaccine dose (i.e. no dose up to 42 days from first dose) among persons who had at least 42 days of follow-up from the first dose.

	Missed Second Dose	Odds Ratio	Adjusted* Odds Ratio
**All persons**	3.63		
**Vaccine Type**			
Pfizer	3.22	1	1
Moderna	3.97	1.24(1.20–1.28)	1.19(1.15–1.23)
**Sex**			
Female (%)	5.74	1	1
Male (%)	3.54	0.60(0.57–0.64)	0.91(0.85–0.98)
**Age (years)**			
18 to <50	10.83	1	1
50 to <60	7.03	0.62(0.58–0.67)	0.64(0.59–0.69)
60 to <65	4.76	0.41(0.38–0.45)	0.44(0.40–0.48)
65 to <70	3.55	0.30(0.28–0.33)	0.33(0.31–0.36)
70 to <75	3.54	0.30(0.28–0.32)	0.33(0.31–0.35)
75 to <80	2.72	0.23(0.22–0.25)	0.24(0.23–0.26)
80 to <85	3.19	0.27(0.25–0.29)	0.29(0.27–0.31)
85 to <90	2.93	0.25(0.23–0.27)	0.27(0.25–0.29)
≥90	3.88	0.33(0.31–0.36)	0.36(0.34–0.40)
**Race**			
White	3.57	1	1
Black	3.7	1.04(0.99–1.08)	0.80(0.76–0.84)
Asian	4.51	1.27(1.09–1.49)	0.97(0.82–1.13)
American Indian/Alaska Native	5.82	1.67(1.43–1.96)	1.34(1.14–1.57)
Pacific Islander/ Native Hawaiian	3.98	1.12(0.95–1.32)	0.99(0.84–1.18)
Declined/Unknown/Missing	3.79	1.06(1.00–1.13)	1.02(0.95–1.09)
**Ethnicity**			
Non-Hispanic	3.63	1	1
Hispanic	3.59	0.99(0.92–1.06)	0.95(0.89–1.03)
Declined/Unknown/Missing	3.61	0.99(0.92–1.07)	0.95(0.87–1.04)
**Urban/Rural**			
Rural/Highly rural	4.27	1	1
Urban	3.08	0.71(0.69–0.73)	0.80(0.78–0.83)
Missing	4.81	1.13(0.96–1.34)	1.15(0.97–1.37)
**Geographical Federal Region** *			
1	3.38	0.89(0.82–0.97)	0.94(0.87–1.02)
2	2.74	0.72(0.67–0.77)	0.78(0.73–0.84)
3	3.34	0.88(0.83–0.94)	0.91(0.86–0.97)
4	3.77	1	1
5	3.08	0.81(0.77–0.85)	0.84(0.80–0.89)
6	5.19	1.40(1.33–1.46)	1.38(1.31–1.44)
7	2.22	0.58(0.54–0.63)	0.56(0.52–0.61)
8	4.82	1.29(1.20–1.40)	1.10(1.02–1.19)
9	3.91	1.04(0.98–1.10)	1.08(1.02–1.15)
10	3.17	0.84(0.77–0.91)	0.80(0.74–0.87)
**VISN**			
1	3.56	1.87(1.70–2.05)	1.79(1.63–1.97)
2	2.9	1.51(1.37–1.66)	1.50(1.36–1.65)
4	2.66	1.38(1.26–1.52)	1.38(1.26–1.52)
5	4.13	2.18(1.97–2.42)	2.01(1.81–2.22)
6	6.47	3.51(3.25–3.78)	3.28(3.03–3.54)
7	5.23	2.80(2.59–3.02)	2.63(2.43–2.85)
8	1.94	1	1
9	4.45	2.36(2.17–2.56)	2.12(1.95–2.31)
10	2.85	1.48(1.36–1.62)	1.45(1.33–1.58)
12	2.43	1.26(1.14–1.39)	1.27(1.15–1.40)
15	2.14	1.11(0.99–1.23)	1.04(0.93–1.15)
16	5.45	2.92(2.72–3.14)	2.67(2.48–2.87)
17	5.25	2.81(2.60–3.03)	2.53(2.35–2.74)
19	5.02	2.68(2.45–2.93)	2.32(2.12–2.55)
20	3.1	1.62(1.47–1.78)	1.45(1.31–1.59)
21	4.09	2.16(1.98–2.35)	2.06(1.89–2.25)
22	3.79	2.00(1.84–2.17)	1.92(1.76–2.08)
23	3.86	2.03(1.87–2.20)	1.74(1.61–1.89)
**BMI (kg/m** ^**2**^ **)**			
<18.5	5.6	1.58(1.37–1.83)	1.57(1.35–1.82)
18.5 to <25	3.62	1	1
25 to <30	3.47	0.96(0.92–1.00)	0.94(0.90–0.98)
30 to <35 (Obese I)	3.61	1.00(0.95–1.04)	0.93(0.89–0.98)
35 to <40 (Obese II)	3.89	1.08(1.02–1.14)	0.95(0.89–1.01)
≥40 (Obese III)	4.4	1.23(1.14–1.32)	1.02(0.95–1.11)
**Charlson Comorbidity Index (CCI)**			
0	4.25	1	1
1	4.09	0.96(0.91–1.01)	1.01(0.96–1.07)
2	3.57	0.83(0.79–0.88)	0.96(0.90–1.01)
3	3.39	0.79(0.75–0.84)	0.92(0.87–0.98)
4	3.24	0.75(0.71–0.80)	0.88(0.83–0.94)
5–6	3.33	0.78(0.74–0.82)	0.92(0.87–0.98)
7–8	3.41	0.80(0.75–0.85)	0.95(0.89–1.01)
≥9	3.27	0.76(0.72–0.81)	0.92(0.86–0.98)
**Diabetes**			
No	3.73	1	1
Yes	3.46	0.93(0.90–0.96)	1.00(0.96–1.03)
**Chronic Kidney Disease**			
No	3.67	1	1
Yes	3.44	0.94(0.90–0.97)	1.02(0.98–1.07)
**Congestive heart failure**			
No	3.63	1	1
Yes	3.69	1.02(0.96–1.08)	1.09(1.03–1.15)
**COPD**			
No	3.66	1	1
Yes	3.52	0.96(0.92–1.00)	1.01(0.97–1.06)
**Tested Positive Before December 11, 2020**			
No	3.6	1	1
Yes	5.19	1.47(1.34–1.61)	1.39(1.27–1.53)
**Percent Moderna vs Pfizer-BioNTech by station**			
<5%	2.31	1	1
5–25%	3.59	1.57(1.47–1.68)	1.74(1.61–1.88)
26–55%	3.22	1.40(1.31–1.50)	2.28(2.11–2.47)
56–95%	3.74	1.64(1.53–1.75)	2.26(2.08–2.46)
>95%	4.34	1.92(1.80–2.04)	2.09(1.94–2.26)

*Simultaneously adjusted for type of vaccine (Moderna vs. Pfizer-BioNTech), age, sex, race, ethnicity, urban/rural, BMI, VISN, CCI, and proportion of Moderna vs. Pfizer-BioNTech doses by station.

### Predictors of vaccine type: Moderna versus Pfizer-BioNTech

Older age was significantly and progressively associated with higher likelihood of receiving the Moderna rather than the Pfizer-BioNTech vaccine (Table 4). Hispanic persons (adjusted odds ratio [AOR] 1.18, 95% CI 1.17–1.20) were more likely to receive the Moderna vaccine, whereas Black persons (AOR 0.73, 95% CI 0.73–0.74) and those living in urban areas (AOR 0.53, 95% CI 0.52–0.53) were less likely to receive the Moderna vaccine. Receipt of the Moderna vaccine varied substantially and significantly by geographical region from 47.78% in Federal Region 10 to 75.99% in Federal Region 8. There was substantial variability by VISN ranging from 48.58% Moderna vaccine in VISN 5 to 61.09% in VISN 19. There was even greater variability by medical center ranging from 0% to 100%. Individual comorbidities or the CCI did not have high magnitude associations with type of vaccine.

**Table 4:** Associations of selected factors with receipt of Moderna versus Pfizer COVID-19 vaccination.

	Proportion who received Moderna (vs Pfizer)	Odds Ratio	Adjusted* Odds Ratio
**All persons**	56.1		
**Sex**			
Female (%)	51.2	1	1
Male (%)	56.4	1.20(1.19–1.22)	1.02(1.00–1.04)
**Age (years)**			
18 to <50	45.49	1	1
50 to <60	49.64	1.17(1.15–1.19)	1.18(1.15–1.21)
60 to <65	53	1.33(1.30–1.35)	1.52(1.48–1.56)
65 to <70	55.76	1.41(1.39–1.43)	1.77(1.73–1.82)
70 to <75	54.62	1.34(1.32–1.36)	1.62(1.58–1.66)
75 to <80	59.32	1.61(1.58–1.63)	1.92(1.88–1.97)
80 to <85	61.7	1.78(1.75–1.81)	2.13(2.07–2.19)
85 to <90	64.06	1.96(1.93–2.00)	2.50(2.43–2.57)
≥90	62.48	1.83(1.79–1.87)	2.43(2.36–2.52)
**Race**			
White	58.75	1	1
Black	46.48	0.61(0.60–0.61)	0.90(0.89–0.92)
Asian	58.57	1.02(0.99–1.06)	1.17(1.12–1.23)
American Indian/Alaska Native	56.87	0.94(0.90–0.97)	0.99(0.93–1.05)
Pacific Islander/ Native Hawaiian	56.46	0.91(0.88–0.95)	1.02(0.97–1.07)
Declined/Unknown/Missing	52.15	0.77(0.76–0.77)	0.98(0.96–1.00)
**Ethnicity**			
Non-Hispanic	56.29	1	1
Hispanic	58.22	1.08(1.07–1.10)	1.04(1.02–1.06)
Declined/Unknown/Missing	50.45	0.79(0.78–0.80)	0.92(0.90–0.95)
**Urban/Rural**			
Rural/Highly rural	64.34	1	1
Urban	49.04	0.53(0.52–0.53)	0.72(0.71–0.73)
Missing	52.1	0.59(0.57–0.62)	0.72(0.68–0.76)
**Geographical Federal Region** *			
1	56.76	1.00(0.98–1.01)	0.94(0.92–0.96)
2	60.86	1.19(1.17–1.21)	0.60(0.59–0.62)
3	55.22	0.96(0.94–0.97)	0.98(0.95–1.00)
4	56.27	1	1
5	55.37	0.95(0.94–0.96)	1.15(1.13–1.17)
6	47.4	0.70(0.69–0.71)	0.91(0.89–0.93)
7	56.56	1.00(0.98–1.01)	0.36(0.35–0.36)
8	75.49	2.39(2.34–2.44)	1.76(1.71–1.82)
9	59.24	1.14(1.13–1.15)	1.02(1.01–1.04)
10	47.44	0.69(0.68–0.71)	1.31(1.27–1.35)
**VISN**			
1	56.46	1.03(1.02–1.05)	0.81(0.80–0.83)
2	58.52	1.13(1.11–1.15)	0.37(0.36–0.38)
4	57.27	1.13(1.12–1.15)	0.73(0.71–0.75)
5	48.58	0.74(0.73–0.76)	0.49(0.46–0.53)
6	56.46	1.03(1.01–1.05)	0.99(0.97–1.01)
7	53.04	0.93(0.92–0.95)	0.73(0.71–0.75)
8	55.05	1	1
9	59.44	1.23(1.21–1.26)	0.43(0.41–0.46)
10	59.5	1.17(1.15–1.18)	0.53(0.51–0.55)
12	53.19	0.94(0.92–0.95)	0.80(0.77–0.82)
15	57.74	1.12(1.10–1.14)	0.55(0.54–0.56)
16	57.2	1.10(1.09–1.12)	0.89(0.86–0.91)
17	57.46	1.08(1.06–1.10)	0.60(0.58–0.61)
19	61.09	1.30(1.27–1.32)	1.19(1.15–1.23)
20	45.9	0.69(0.68–0.70)	1.03(0.99–1.06)
21	53.5	0.95(0.93–0.96)	0.91(0.89–0.93)
22	57.49	1.11(1.09–1.13)	0.93(0.90–0.96)
23	57.94	1.10(1.08–1.12)	0.50(0.49–0.52)
**BMI (kg/m** ^**2**^ **)**			
<18.5	55.58	0.97(0.93–1.00)	0.95(0.90–1.01)
18.5 to <25	56.39	1	1
25 to <30	56.57	1.01(1.00–1.02)	1.03(1.02–1.04)
30 to <35 (Obese I)	55.71	0.98(0.97–0.99)	1.02(1.00–1.03)
35 to <40 (Obese II)	55.34	0.97(0.96–0.98)	1.03(1.02–1.05)
≥40 (Obese III)	55.43	0.98(0.97–1.00)	1.04(1.02–1.07)
**Charlson Comorbidity Index (CCI)**			
0	52.42	1	1
1	56.08	1.15(1.14–1.16)	1.05(1.03–1.07)
2	57.4	1.20(1.19–1.22)	1.04(1.03–1.06)
3	57.84	1.22(1.21–1.24)	1.03(1.01–1.04)
4	58.05	1.23(1.21–1.24)	1.01(0.99–1.03)
5–6	58.3	1.24(1.23–1.25)	0.99(0.97–1.01)
7–8	57.31	1.18(1.17–1.20)	0.93(0.91–0.95)
≥9	54.7	1.06(1.05–1.08)	0.83(0.82–0.85)
**Diabetes**			
No	55.32	1	1
Yes	57.54	1.09(1.08–1.09)	1.01(1.00–1.02)
**Chronic Kidney Disease**			
No	55.68	1	1
Yes	58.75	1.12(1.11–1.13)	0.98(0.97–1.00)
**Congestive heart failure**			
No	56.11	1	1
Yes	55.86	0.98(0.96–0.99)	0.86(0.84–0.88)
**COPD**			
No	55.44	1	1
Yes	59.45	1.17(1.16–1.18)	1.00(0.98–1.01)
**Tested Positive Before December 11, 2020**			
No	56.14	1	1
Yes	54.05	0.93(0.90–0.95)	0.84(0.81–0.87)

* Simultaneously adjusted for age, sex, race, ethnicity, urban/rural, BMI VISN, CCI, and proportion of Moderna vs. Pfizer doses by station.

## Discussion

In this study of 5,766,638 Veterans who received care in the VA healthcare system, 1,569,101 (27.2%) were vaccinated from December 11, 2020 to March 9, 2021. Factors independently associated with receipt of vaccination during this early period included older age, higher comorbidity burden, a number of individual comorbid conditions (CHF, CKD, COPD and diabetes), higher body mass index (BMI) category, Black or Asian (vs. White) race, Hispanic (vs. non-Hispanic ethnicity), urban (vs. rural) residence, and geographical region in the US, while AI/AN race (vs. White) was associated with lower vaccination rate. Adherence to the receipt and recommended timing of the second vaccine dose was remarkably high for both Moderna and Pfizer-BioNTech vaccines. Factors associated with higher likelihood of missing the second dose included younger age, AI/AN race, female sex, rural location, geographical region and receiving the Moderna rather than the Pfizer-BioNTech vaccine.

The CDC phases of COVID-19 vaccination prioritization strongly emphasized age (≥75 years in phase 1b and 65–74 years in phase 1c) and high-risk medical conditions (allocated to phase 1c in those aged 16–64 years) [1]. Consistent with these recommendations, our results demonstrate the highest vaccination rate during the observation period in persons ≥75 years of age. Among this age group, the highest vaccination rate was in those 75 to <80 years of age (45.74%) with a slight decline in even older age groups. Vaccination rates were also very high in those 65 to <70 (35.97%) and 70 to <75 (38.33%) years old, who correspond to phase 1c. Individual comorbid conditions that have been associated with risk of adverse outcomes in SARS-CoV-2, such as diabetes, CHF, COPD and CKD, as well as overall comorbidity burden were also strongly associated with vaccination.

Studies based on national US vaccination data suggested that Black and Hispanic persons have received smaller shares of vaccinations compared to their shares of the population in most states of the US [24, 25]. However, interpretation of these results is limited by the high percentage of records with unknown or missing race/ethnicity information (almost 50%), the unknown proportions of healthcare personnel among early vaccine recipients, and the lack of adjustment for comorbid conditions and other potential confounders. Our results among VA enrollees excluding VA employees (i.e. excluding most healthcare workers) show that although Black, Asian and Hispanic persons have slightly lower unadjusted rates of vaccination than White or non-Hispanic persons, they were actually more likely to receive vaccination after adjustment for other sociodemographic factors and comorbid conditions. In contrast, AI/AN persons had the lowest vaccination rates of any racial/ethnic group, an association that persisted after adjustment for baseline characteristics (AHR 0.83, 95% 0.82–0.85).

Person living in urban (vs. rural) areas were significantly more likely to undergo vaccination in both unadjusted and adjusted analyses. Cold storage requirements for Pfizer and Moderna vaccines and distance to vaccination centers may contribute to lower vaccination rates in rural areas. Additionally, there was substantial variation in vaccination by both federal region and VISN, which likely reflects regional logistical limitations in access to vaccines and ability to use them quickly.

Only 3.6% of Moderna or Pfizer-BioNTech first vaccine dose recipients failed to receive the second dose within 42 days. This is likely an even higher completion rate than was reported in study based on the US population as of February 14, 2021 (88% completion, 8.6% no second dose but still within allowable interval and 3.4% missed dose), although results in that study were not limited to those with at least 42 days of follow-up [26]. Our results suggest that the most profound variation is related to age. Older age was associated with a dramatic reduction in the likelihood of missing the second dose from 10.83% in 18 to <50 year-olds to 2.72% in 75 to <80 year-olds (AOR 0.24, 95% CI 0.23–0.26), with a slight increase in missed second doses in even older age groups. Future efforts should focus on understanding the reasons for higher rates of missed second doses in younger persons and implementing interventions to improve adherence. Racial/ethnic minority groups did not have lower adherence to second dose vaccination, with the exception of AI/AN persons who were significantly more likely to miss the second dose. Thus, AI/AN persons were both less likely to get vaccinated and less likely to receive the required second dose. There was also substantial geographical variability in adherence to second vaccine dosing, much more pronounced at the VISN rather than the federal region level. This suggests that institutional as well as geographical issues likely affect second dose adherence. The fact that vaccination with Moderna (vs. Pfizer-BioNTech) was associated with slightly higher likelihood of missed second dose, requires further confirmation: we did not explicitly apply methods of clinical trial emulation and causative inference in our study that was not specifically designed to compare the two vaccines.

Geographical differences in use of Moderna versus Pfizer vaccines likely reflect the numbers of each vaccine that were distributed to each VA VISN and medical center. The fact that that rural areas were associated with lower use of Pfizer-BIoNTech may be related to the more stringent cold storage requirements for Pfizer-BioNTech (-70C) versus Moderna (-20C). The very strong association of older age with Moderna use is hard to explain, may be due to unmeasured confounding and requires additional confirmation. There was little or no flexibility for VA enrollees to select their preferred vaccine during the study period.

Persons previously testing positive with SARS-CoV-2 were slightly more likely to be vaccinated but significantly more likely to miss the second vaccine dose. The requirement for a second dose should be emphasized in persons with prior infection.

Limitations of our study include lack of data on any additional vaccinations that might have been performed among VA enrollees outside of the VA healthcare system. Our results apply to VA enrollees who are predominantly male and tend to have more comorbid conditions and adverse social determinants of health, but also have access to comprehensive, high quality healthcare that has been shown to attenuate some racial and ethnic disparities [27]. We did not have access to any side effects or adverse reactions experienced after vaccination to test whether they were associated with second dose adherence.

We described **s**ociodemographic and clinical factors that may be used to target vaccination efforts and to further improve adherence to second vaccine dosing. Continued monitoring of vaccination uptake, type of vaccine used and adherence to dosing requirements will be necessary as vaccine efforts expand even further.

## Supporting information

S1 Fig a. Graphical assessment of the proportional hazards assumption using log(-log) plots for Charlson Comorbidity Index (CCI) categories. b. Graphical assessment of the proportional hazards assumption using log(-log) plots for Chronic Kidney Disease (CKD) categories.
(TIF)
