# Supplementary figures and images for: Factors associated with early receipt of COVID-19 vaccination and adherence to second dose in the Veterans Affairs healthcare system
Source: PLoS One. 2021 Dec 1;16(12):e0259696. doi: 10.1371/journal.pone.0259696 (PMC8635372; doi:10.1371/journal.pone.0259696)

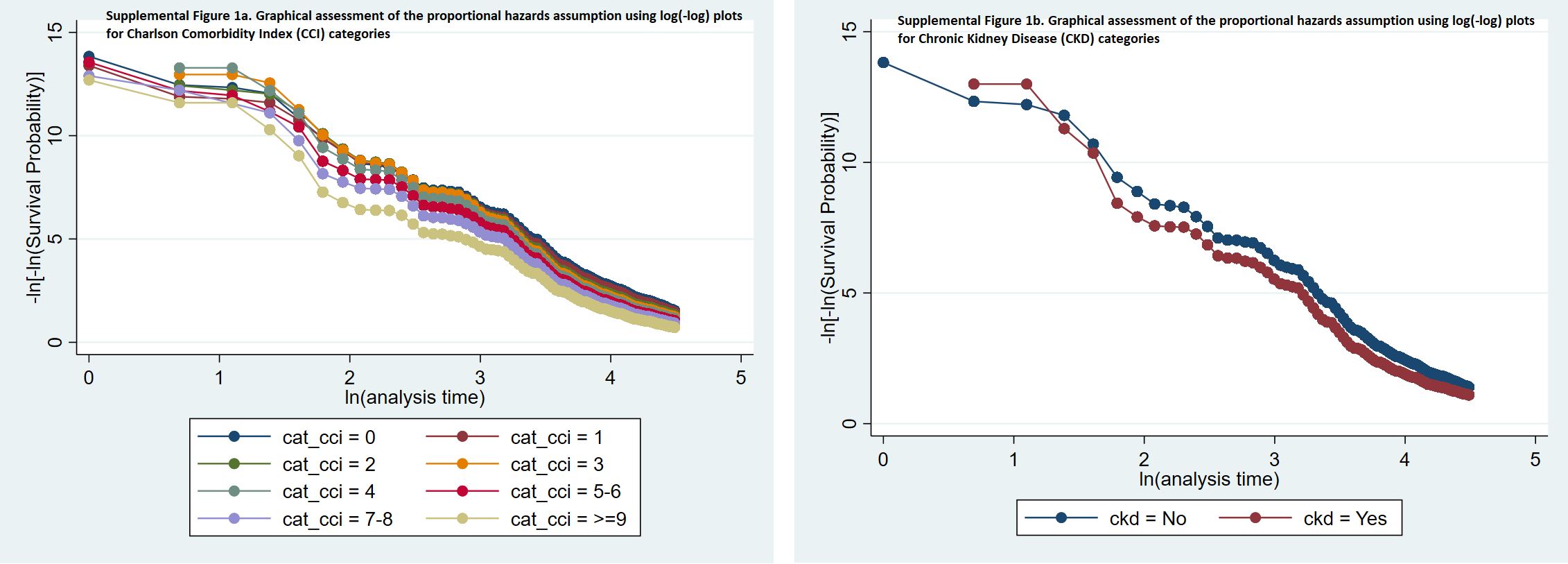

Supplement: S1 Fig — a. Graphical assessment of the proportional hazards assumption using log(-log) plots for Charlson Comorbidity Index (CCI) categories. b. Graphical assessment of the proportional hazards assumption using log(-log) plots for Chronic Kidney Disease (CKD) categories. (TIF) [file pone.0259696.s001.tif]
